# Resolvin D1 improves bleomycin-induced alveolar maturation arrest in newborn rats

**DOI:** 10.1038/s41598-025-12739-4

**Published:** 2025-08-05

**Authors:** Jun Shinohara, Tohru Ogihara, Shigeo Yamaoka, Yutaro Kawamura, Daisuke Nishioka, Kazumi Honda, Yumiko Yoshimura, Akira Ashida

**Affiliations:** 1 Division of Neonatology, Department of Pediatrics, Osaka Medical and Pharmaceutical University, 2-7, Daigaku-Machi, TakatsukiOsaka, 569-0801 Japan; 2 Department of Medical Statistics, Osaka Medical and Pharmaceutical University, Research & Development Center, Takatsuki, Osaka Japan

**Keywords:** Bronchopulmonary dysplasia, Resolvin D1, Insulin-like growth factor-1, Tenascin-C, Specialized pro-resolving mediators, Molecular biology, Diseases, Molecular medicine

## Abstract

Sustained, non-resolving inflammation is a fundamental mechanism that causes bronchopulmonary dysplasia (BPD). Specialized pro-resolving mediators (SPMs) are attracting attention as the new endogenous anti-inflammatory agents because they facilitate only the resolution phase of inflammation without affecting its acute phase, indispensable for the elimination of noxious microorganisms and damaged tissues. The preventive effects of resolvin D1 (RvD1), an SPM, were analyzed using bleomycin (Bleo)-induced BPD model of neonatal rats. Starting from the postnatal day (PD)-0, Bleo and RvD1 were administered simultaneously. At PD-14, morphological and immunohistochemical analyses, and the expression of key genes suspected to be involved in the Bleo-induced lung damage, were examined. Bleo injection successfully recapitulated the pathological features of BPD, such as alveolar enlargement and simplification with septal thickening. RvD1 effectively inhibited such alveolar dysmorphogenesis with attenuating macrophage infiltration and restoring the arrested capillary growth. It significantly suppressed the Bleo-induced upregulation of insulin-like growth factor-1 (IGF-1), tenascin-C, elastin, and anillin. The changes in IGF-1 protein expression in the bronchial and alveolar epithelia were confirmed by immunostaining. RvD1 effectively improves Bleo-induced model of alveolar maturation arrest of BPD. The supplementation of SPMs including RvD1 seems to be a promising treatment for the infants with BPD.

## Introduction

Despite remarkable progress in neonatal medicine, bronchopulmonary dysplasia (BPD), a chronic respiratory disease specific to preterm infants, has not yet been overcome^1^. BPD remains a major cause of morbidity for extremely low gestational age newborns and often leaves adverse sequelae such as respiratory and neurodevelopmental disabilities^2,3^. BPD is a multifactorial clinical syndrome and the cumulative result of superimposed processes of chronic or repetitive injury followed by aberrant repair and disturbed development of the lungs^4,5^. Sustained, non-resolving inflammation is a fundamental mechanism leading to the onset and development of BPD^6^.

The principle of treatment of inflammatory diseases has traditionally been the overall suppression of the inflammation process. BPD treatment follows the same principle with corticosteroids, the representative anti-inflammatory agents, being the mainstay of treatment even today, despite their unavoidable side-effects and limited efficacy^6,7^. Fundamentally, inflammation is an adaptive immune response necessary to survive and restore homeostasis against various noxious stimuli and conditions^6,8,9^. Therefore, the inflammation should not be uniformly suppressed throughout its entire course.

Since the discovery of specific lipid mediators in resolving exudates^10^, a major paradigm shift has occurred surrounding the concept of inflammation^11^. The resolution of inflammation, earlier considered passive, is now recognized as a biosynthetically active process regulated by a superfamily of endogenous chemical mediators, including peptides, gases, and lipids, collectively known as specialized pro-resolving mediators (SPMs)^12^. SPMs include polyunsaturated fatty acid-derived autacoids, such as resolvins, protectins, and maresins synthesized from omega-3 essential fatty acids, as well as lipoxins from omega-6 fatty acids. SPMs enhance only the resolution process of inflammation without interfering with its acute phase^13,14^. Unlike conventional anti-inflammatory modulators, therefore, SPMs appear to be ideal anti-inflammatory agents.

Chronic inflammation is the fundamental driver of many diseases^12^. In patients with chronic and persistent inflammatory diseases, such as chronic obstructive pulmonary disease, rheumatoid arthritis, systemic lupus erythematosus, and neurodegenerative diseases, a decrease in multiple SPMs has been observed, suggesting an underlying failure of resolution process^11^. The administration efficacy of various SPMs has been reported in animal models of inflammation and microbial infection, including lung diseases such as allergic airway disease, acute respiratory distress syndrome, and pneumonia^13,14^. As for BPD, a few animal studies have examined the effects of SPMs; all were hyperoxia-induced murine BPD models and showed a favorable effect of resolvin D1 (RvD1), lipoxin A_4_, and protectin^15–18^.

RvD1 [7S,8R,17S-trihydroxy-4Z,9E,11E,13Z,15E,19Z-docosahexaenoic acid] is a potent SPM first described in 2002^19^. It is produced by sequential reactions of 15-lipoxygenase and 5-lipoxygenase on docosahexaenoic acid. It limits the neutrophil and monocyte recruitment, induces their apoptosis, enhances clearance of debris and apoptotic neutrophils (efferocytosis) by the macrophages, reduces the production of pro-inflammatory mediators in various inflammation-related cells, and modulates the phenotypic propensities of macrophages (Supplementary Fig. S1 online)^11,13,14,20,21^.

Our aim was to investigate the efficacy of RvD1 in a murine model of BPD and determine the underlying molecular mechanism. We used the bleomycin (Bleo) loading model for newborn rats because our previous paper confirmed that it closely resembles the histopathology of human BPD, showing enlarged, fewer, simplified alveolar structures, thickened alveolar walls, and reduced pulmonary vasculature^22^. In fact, it is often used as a BPD model^23–26^. Additionally, several reports show the efficacy of SPMs, including RvD1 in the Bleo-induced idiopathic pulmonary fibrosis (IPF) model^27–30^, which is among the reasons that led us to decide on using the Bleo model.

## Results

Of the 48 pups studied, one from the Bleo group died during this study.

### RvD1 fails to prevent reduced somatic growth by Bleo

Mean weight gain from birth to postnatal day (PD)-14 (the end of the experiments) was significantly reduced following Bleo administration, and RvD1 did not prevent the reduction (Bleo, 20.0 ± 2.2 g and Bleo + RvD1, 20.3 ± 2.4 vs. Control, 25.9 ± 2.8, *p* < 0.001, respectively). However, RvD1 alone exhibited no influence on the weight gain (Supplementary Fig. S2 online).

### RvD1 restores Bleo-induced alveolar simplification and septal wall thickness

**Fig. 1:**
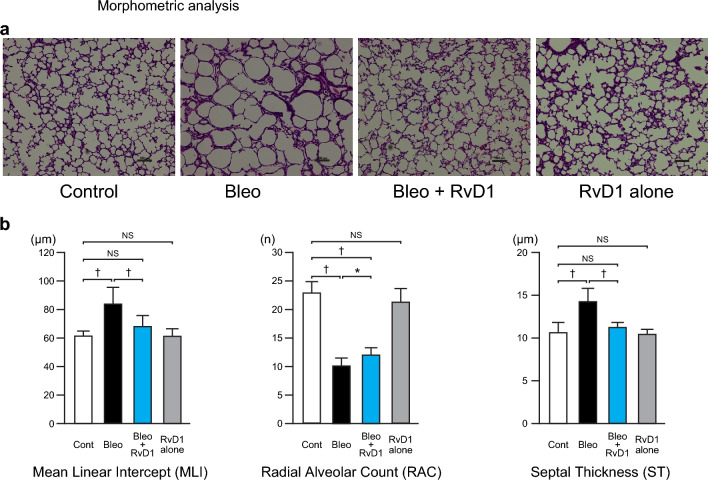
RvD1 restores Bleo-induced alveolar simplification and septal wall thickness. a Representative lung histology stained with hematoxylin and eosin. Magnification, 100 × ; Scale bar, 100 μm. b Mean linear intercept (MLI), radial alveolar count (RAC), and septal thickness (ST). White bar: Control group, black bar: Bleo group, blue bar: Bleo + RvD1 group (500 ng daily for 10 days), gray bar: RvD1 alone group (500 ng RvD1 alone daily for 10 days). Mean ± SD. N = 5–8/group.* *p* <0.05, † *p* < 0.001, NS: not significant. The Bleo group displayed impaired alveolarization, increased MLI and ST, and decreased RAC compared to that of the Control group. Treatment with RvD1 improved the alveolar structure and significantly improved the MLI, RAC, and ST values. RvD1 alone had no apparent effect on histological or morphometric analyses.

We assessed whether our Bleo model is suitable for studying BPD and whether RvD1 inhibits the morphological changes caused by Bleo administration. As shown in Fig. 1a, Bleo treatment resulted in enlarged alveoli, decreased alveolar complexity, and thickened alveolar walls compared to those in the Control group. Both mean linear intercept (MLI) and septal thickness (ST) were significantly increased, and radial alveolar count (RAC) was significantly reduced in the Bleo group as compared to the Control group (Fig. 1b). Therefore, we confirmed that our model correctly reproduced the alveolar simplification and septal wall thickness seen in infants with BPD, as in our previous report^22^.

RvD1 improved the abnormalities in the alveolar structure, bringing them closer to those in the Control group (Fig. 1a). Through morphometry (Fig. 1b), we observed that RvD1 effectively reduced MLI and ST (MLI: Bleo + RvD1, 67.8 ± 7.8 μm vs. Bleo, 83.5 ± 11.8, *p* < 0.001, Cohen’s *d* = 1.9; ST: Bleo + RvD1, 11.2 ± 0.6 μm vs. Bleo, 14.2 ± 1.5, *p* < 0.001, Cohen’s *d* = 2.8), and increased RAC (Bleo + RvD1, 12.0 ± 1.3 vs. Bleo, 10.1 ± 1.4, *p* < 0.05, Cohen’s *d* = 1.5). In addition, RvD1 fully restored the Bleo-induced increase in MLI and ST to the levels observed in the Control group. RvD1 alone did not affect alveolar formation.

### RvD1 inhibits macrophage influx

The Bleo model shows accumulation of neutrophils, macrophages, and monocytes in the bronchoalveolar lavage fluid and lung tissue^28,30,31^. However, the accumulation patterns vary widely and are primarily influenced by the number of days subsequent to Bleo administration and intervention method employed. Amongst, macrophages play a pivotal role in resolving inflammation and constitute the primary target of SPMs during this process (Supplementary Fig. S1 online). Although neutrophil staining with Ly-6G antibodies was attempted, these cells were present in very low quantities, including in the Bleo-only group. Thus, we focused on comprehensive examination of macrophages.

**Fig. 2:**
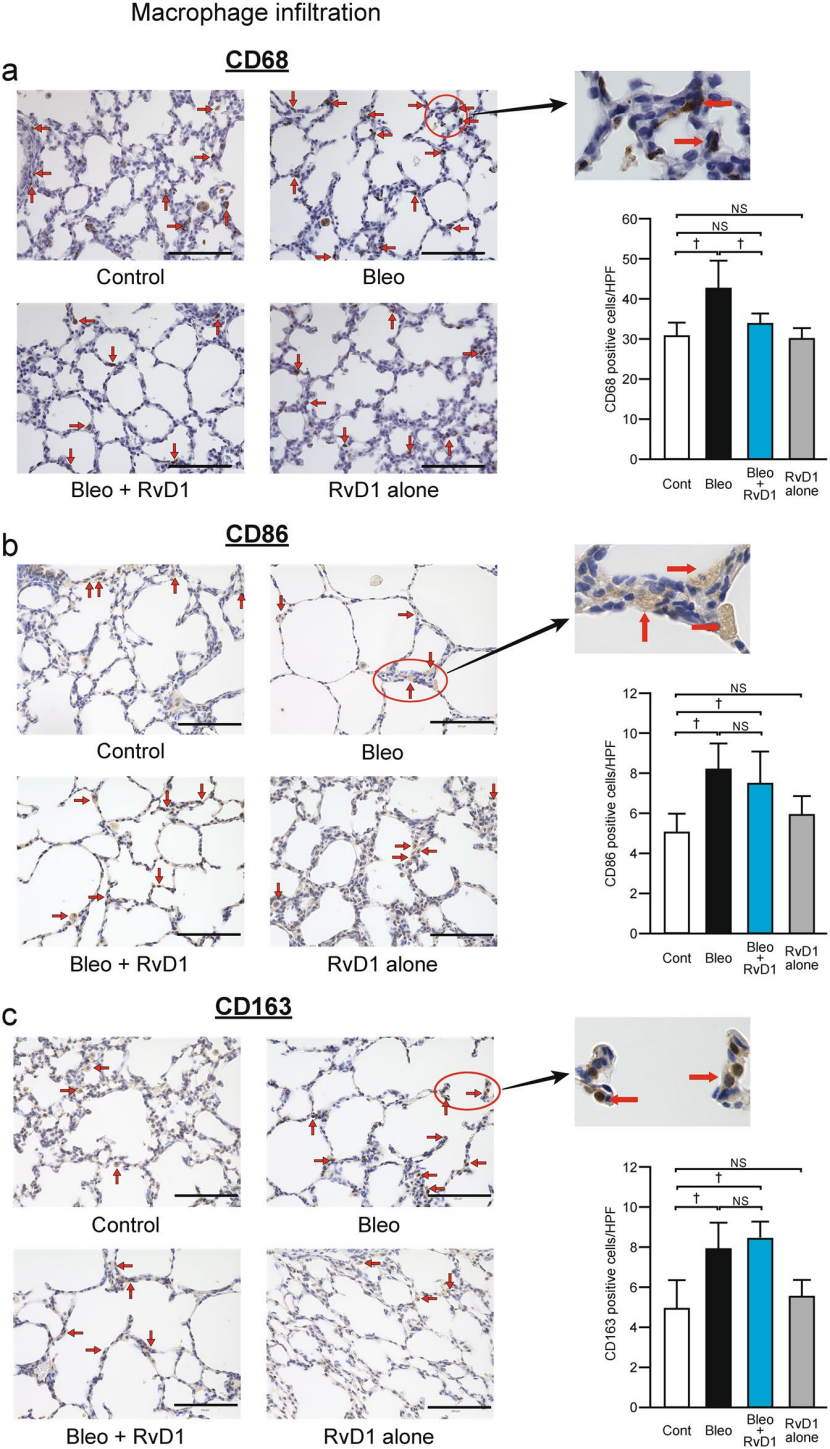
RvD1 inhibits macrophage influx induced by Bleo. The left-hand panels show representative immunostaining with a CD68 (common to each macrophage subtype), b CD86 (relatively specific to ‘M1-like’ phenotype), and c CD163 (relatively specific to ‘M2-like’ phenotype) antibodies, respectively. Red arrows indicate corresponding antibody-positive macrophages. Magnification, 100 × ; Scale bar, 100 μm. The right-hand graphs indicate the number of each left antibody-positive macrophages in the HPF (magnification 100 ×). White bar, Control group; black bar, Bleo group; blue bar, Bleo + RvD1 group; gray bar, RvD1 alone group. Mean ± SD. N = 5–8/group. † *p* < 0.001, NS: not significant. Bleo administration resulted in macrophage infiltration; however, RvD1 significantly reduced the number of macrophages to that of the control level. Both M1 and M2 phenotypes were increased by Bleo and RvD1 did not significantly influence their changes. HPF, high-power field.

In exploring macrophage behavior, we first examined the infiltration of macrophages as a whole, using CD68, an antigen common to each macrophage subtype. As shown in Fig. 2a, the numbers of total macrophages were significantly higher in the Bleo group than those in the Control group (Bleo, 42.5 ± 6.8 vs. Control, 30.7 ± 3.3, *p* < 0.001). When RvD1 was simultaneously administered with Bleo, it returned to the control level (33.7 ± 2.6, not significant (NS) vs. Control). These results show that RvD1 effectively inhibited overall macrophage infiltration induced by the Bleo injection. To ascertain whether macrophage polarization associated with inflammatory resolution developed, additional investigation was performed using CD86 antibodies, which are mainly expressed in pro-inflammatory ‘M1-like’ phenotype, and CD163 antibodies, which are relatively specific to anti-inflammatory, pro-resolving ‘M2-like’ phenotype^32^. Both phenotypes were significantly increased by Bleo treatment (CD86: Bleo, 8.2 ± 1.3 vs. Control, 5.0 ± 0.9, *p* < 0.001; CD163: Bleo, 7.9 ± 1.3 vs. Control, 4.9 ± 1.4, *p* < 0.001) but RvD1 had no effect on these changes (Fig. 2b, c).

### RvD1 cancels Bleo-induced capillary growth impairment

**Fig. 3:**
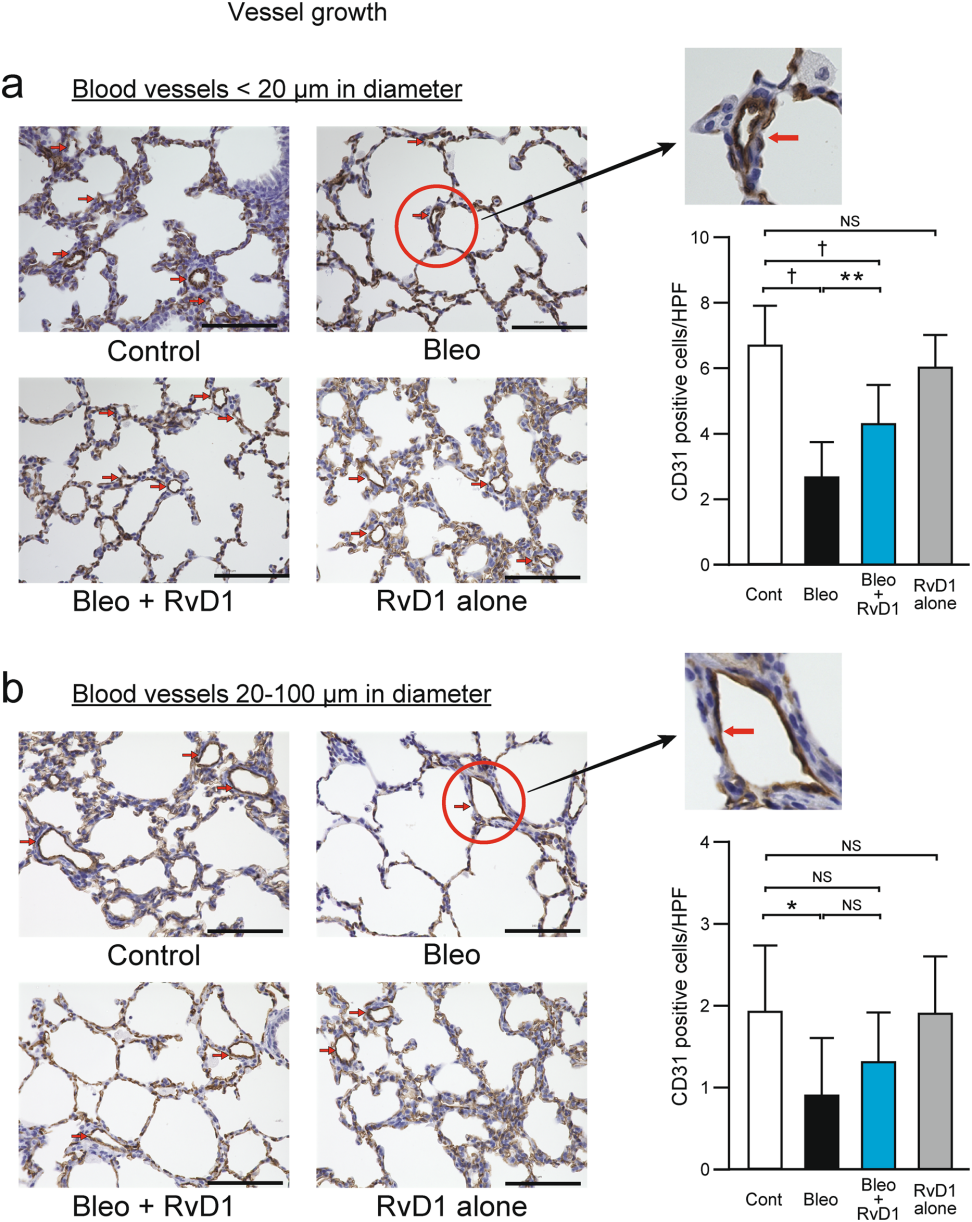
RvD1 restores capillary growth impairment induced by Bleo. The left-hand panels show representative immunostaining of the vascular endothelial cells as an index of vascularization in capillaries < 20 µm in diameter (**a**) and small vessels of 20–100 µm in diameter (**b**). Red arrows indicate CD31-positive endothelial cells. Magnification, 100 × ; Scale bar, 100 μm. The right-hand graphs indicate the number of CD31-positive endothelial cells with diameter shown in the left in the HPF (magnification 100 ×). White bar, Control group; black bar, Bleo group; blue bar, Bleo + RvD1 group; gray bar, RvD1 alone group. Mean ± SD. N = 5–8/group. * *p* < 0.05, ** *p* < 0.01, † *p* < 0.001. Bleo administration significantly interfered with vascular development. RvD1 restored the number of capillary (< 20 µm in diameter) endothelial cells but did not reach the control levels. HPF, high-power field.

During embryogenesis of the lung, alveolar growth occurs parallel to vascular development. Abnormal vascular development is often observed in patients with BPD^33^. As shown in Fig. 3a, when Bleo coexisted during lung development, development of capillaries less than 20 µm in diameter was significantly suppressed as assessed by the number of CD31-positive vascular endothelial cells (Bleo, 2.6 ± 1.1 vs. Control, 6.7 ± 1.2, *p* < 0.001). RvD1 restored the number of endothelial cells (4.3 ± 1.2, *p* < 0.01 vs. Bleo); however, the number did not reach that of the control levels. A similar trend was observed in microvessels larger than capillaries, but RvD1 was unable to significantly abolish the Bleo-induced inhibition of vascular development (Fig. 3b).

### Bleo loading does not elicit a substantial increase in myofibroblasts within this particular model

**Fig. 4:**
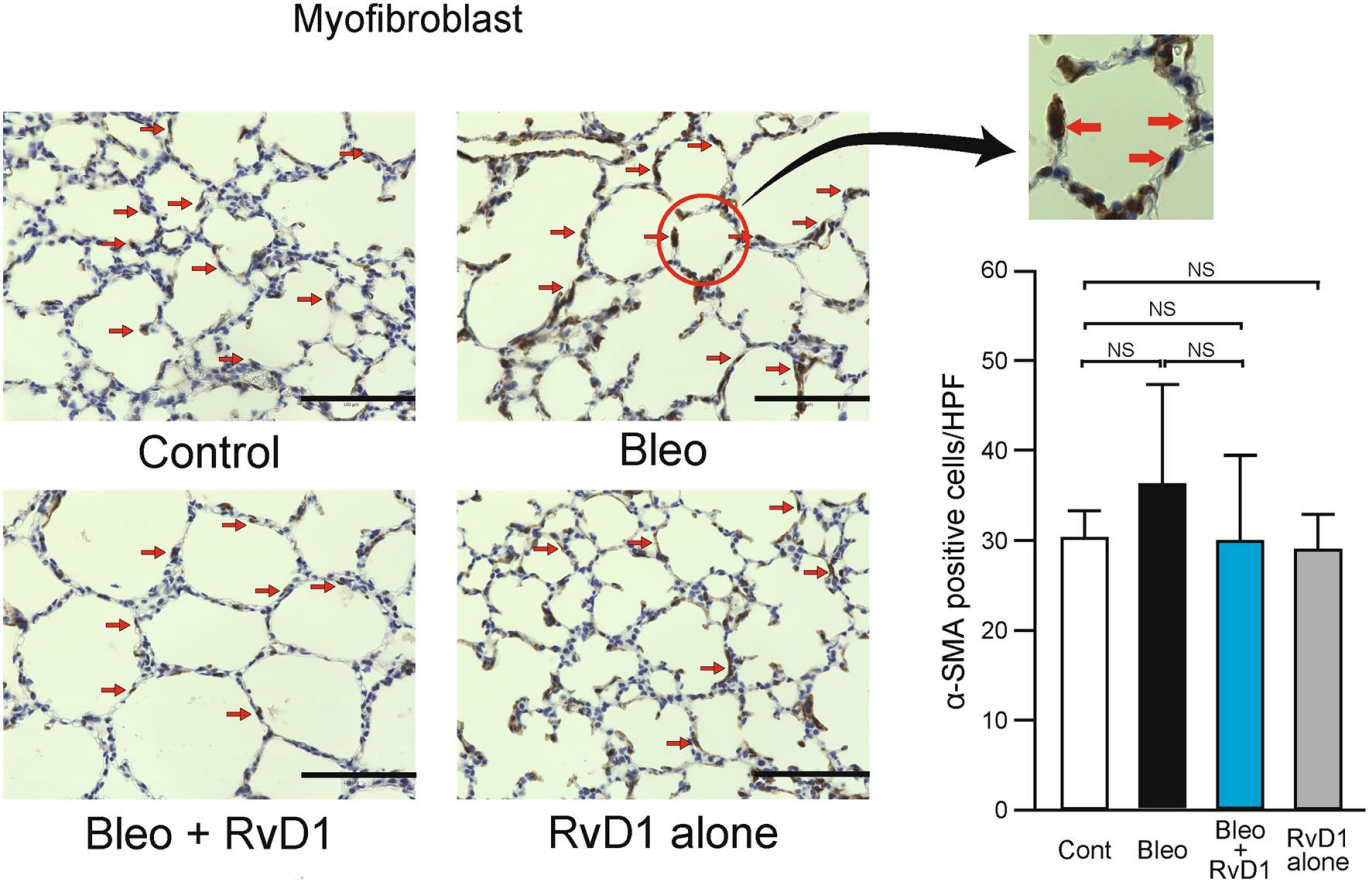
Bleo loading does not elicit a substantial increase in myofibroblasts within this particular model. The left-hand panels show representative immunostaining of the myofibroblasts as an index of fibrosis. Red arrows indicate anti-α-SMA-positive cells. Magnification, 100 × ; Scale bar, 100 μm. The right-hand graphs indicate the number of anti-α-SMA-positive cells in the HPF (magnification 100 ×). White bar, Control group; black bar, Bleo group; blue bar, Bleo + RvD1 group; gray bar, RvD1 alone group. Mean ± SD. N = 5–8/group. NS: not significant. Bleo induced an increase in myofibroblasts; nevertheless, this increase was not statistically significant. HPF, high-power field.

Increased levels of myofibroblasts have been observed in the Bleo-IPF model, and RvD1 can suppress increases in α-SMA, a marker of myofibroblasts, in this model^30^. In human BPD, myofibroblasts show an aberrant distribution with increased proliferation^34^. Thus, we also examined changes in α-SMA in the current model. As shown in Fig. 4, Bleo induced an increase in myofibroblasts; nevertheless, this increase was not statistically significant.

### Genetic background of Bleo-induced alveolar dysmorphogenesis and its suppression by RvD1

To explore the fundamental mechanisms behind the effects of RvD1, we decided to examine its genetic background. First, we searched for candidate genes to be examined by reviewing the gene expression profiles of bleomycin models^35,36^, patients with BPD^37^/IPF^36,38^, and mammalian lung embryogenesis^39,40^ (Supplementary Table S1 online). From the extensive list of gene sets with significant variations, we selected the common genes mentioned in at least two studies from different fields. From these candidate genes, we first selected 55 genes, listed under “first selection” in Supplementary Table S2 online, according to the following rule; we judged that human BPD, mouse Bleo model, human IPF, and lung development were most closely related to our model in this order. We prioritized the genes that crossed over to each field more frequently in the same order. The fundamental genes commonly involved in cellular homeostasis, including cell division, energy metabolism, and programmed cell death, were excluded, and genes that appeared to be closely related to lung development and fibrosis were selected. Finally, we selected the following six genes: anillin actin-binding protein (ANLN), insulin-like growth factor-1 (IGF-1), phospholipase A2 group IIA (PLA2G2A), a disintegrin and metalloprotease with thrombospondin domains (ADAMTS-12), elastin (ELN), and tenascin C (TNC). ANLN is a conserved scaffolding protein essential for basic cellular function common to all tissues, indispensable for cytokinesis at specific stages of cell division^41^. Therefore, according to the above criteria, it should have been excluded from our analysis; however, it was selected because it had the highest number of hits among the references.

**Fig. 5:**
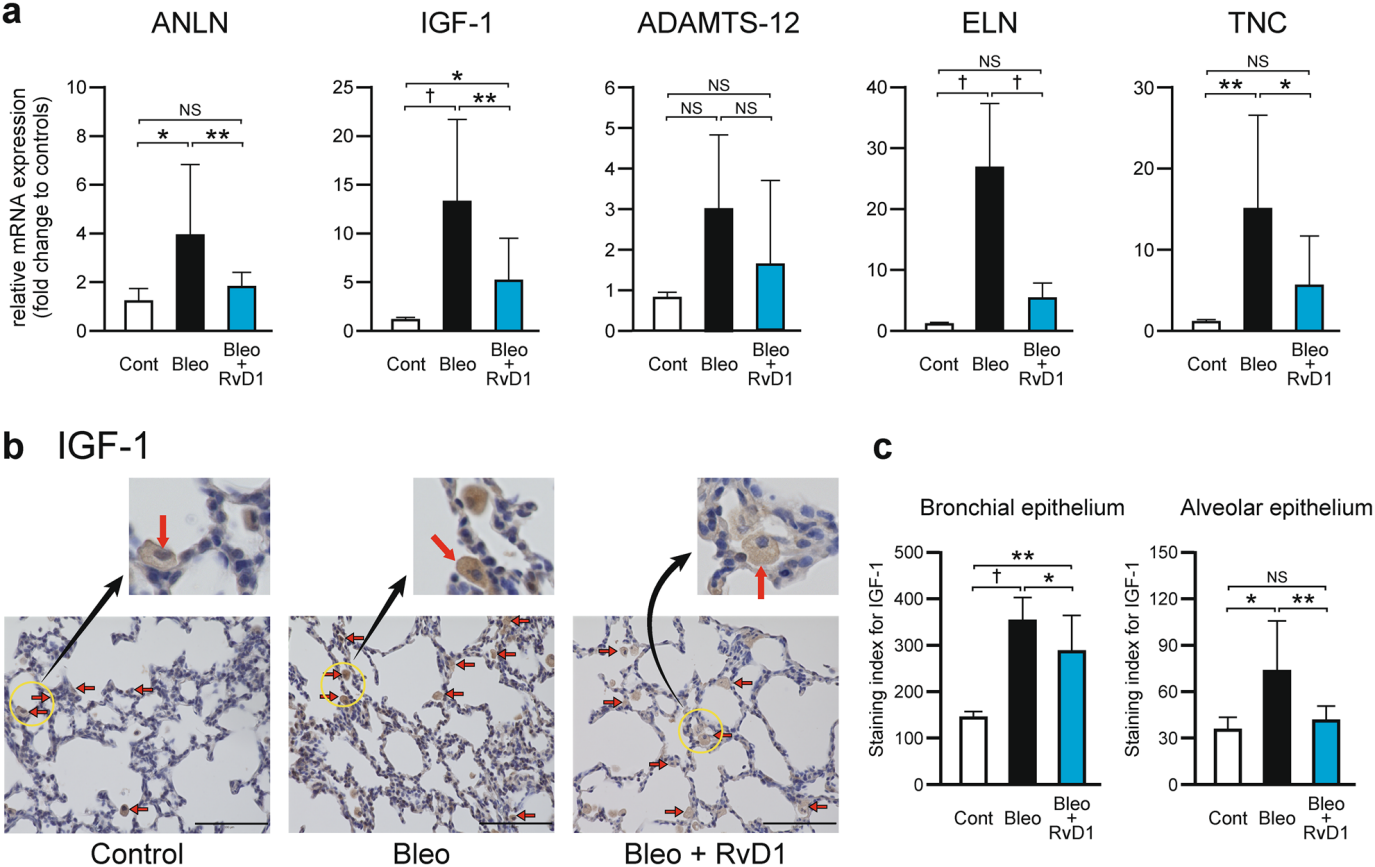
mRNA expression of selected genes possibly involved in the development of Bleo-induced alveolar simplification and IGF-1 protein expression. a Relative mRNA expression of ANLN, IGF-1, ADAMTS-12, ELN, and TNC. Real-time PCR was performed using GAPDH as a standardizing control. The level of expression of each mRNA was calculated using the 2^−ΔΔCT^ method, and the fold change to the control level was displayed. Mean ± SD. N = 5–8/group. * *p* < 0.05, ** *p* < 0.01, † *p* < 0.001, NS: not significant. Bleo upregulated these genes, and RvD1 suppressed the upregulation of the genes other than ADAMTS-12. b Representative immunostaining of IGF-1. Red arrows show IGF-1 expressions in the alveolar type II cells and bronchial epithelium. c The intensity of IGF-1 staining was scored, IGF-1-positive cells were counted, and the extent of IGF-1 expression was evaluated by the staining index (staining intensity × percentage of stained cells). Mean ± SD. N = 5–8/group. * *p* < 0.05, ** *p* < 0.01, † *p* < 0.001, NS: not significant. White bar: Control group; black bar: Bleo group; blue bar: Bleo + RvD1 group. IGF-1-positive cells were significantly increased by Bleo but were restored by RvD1.

Among these, the expression of PLA2G2A was not observed. The relative mRNA expression levels of the remaining five genes on PD-14 are shown in Fig. 5a. All five genes were upregulated by Bleo injection, and except for ADAMTS-12, the differences in expression levels of the other four genes were statistically significant. Furthermore, the Bleo-induced upregulation of mRNA expression levels of these four genes was significantly suppressed by co-treatment with RvD1 (Fig. 5a).

As for IGF-1, it is well recognized as an essential growth factor for lung development^42,43^, however, its mRNA expression status in this study was contradictory. Therefore, we decided to confirm its expression at the protein level using immunohistochemistry. As shown in Fig. 5b, the endothelial epithelium and smooth muscles were not clearly stained. Therefore, the degree of IGF-1 staining in the bronchial and alveolar epithelia was evaluated using a staining index (Fig. 5c). The results showed that Bleo increased IGF-1 protein expression in both the epithelia (bronchial epithelium: Bleo, 352 ± 53 vs. Control, 143 ± 14, *p* < 0.001; alveolar epithelium: Bleo, 73 ± 32 vs. Control, 35 ± 8, *p* < 0.05). To counter the effect of Bleo, RvD1 significantly suppressed the increase in IGF-1, especially in the alveolar epithelium, down to the control level (41 ± 9, NS vs. Control).

## Discussion

In the present study, we confirmed that RvD1 effectively improved Bleo-induced alveolar simplification and septal thickening (Cohen’s *d*: 1.5–2.3, very large effects), partly through attenuation of macrophage infiltration, restoration of arrested capillary growth, and suppression of enhanced gene expression of IGF-1, TNC, ELN, as well as ANLN. This is the first report to show a positive effect of SPMs in a Bleo-induced BPD model. Our demonstration in BPD murine model, other than hyperoxic exposure models^15–18^, has the potential to boost clinical applications of SPMs in the future and become a new treatment for BPD.

The suppression of macrophage infiltration by SPMs including RvD1 has already been reported in the Bleo-induced IPF model^27,28,30^. As for macrophage subtypes, it is known that SPMs promote its polarization from the pro-inflammatory ‘M1-like’ phenotype to the anti-inflammatory, pro-resolving ‘M2-like’ phenotype^14,27^. Our present data shows that both phenotypes were increased by Bleo while RvD1 administration did not significantly influence their changes. The M1 and M2 phenotypes each have multiple discriminative markers, and previous reports have shown that their combination may yield contradictory outcomes, even with exactly the same model^27,30^. If other combinations of specific antibodies were used and/or the examination window was expanded, the process of transitions may be captured.

As represented by the so-called ‘a vascular hypothesis’ of BPD pathogenesis, it is well known that reduction in lung microvascularization is the central feature of BPD pathology, since angiogenesis occurs in parallel with and is closely interrelated with alveolarization^33^. Proteomic analyses using blood samples from preterm infants with BPD showed that alterations of circulating angiogenic proteins in early postnatal period, including angiopoietin-1 (ANG-1) and vascular endothelial growth factor (VEGF), were closely associated with subsequent development of BPD^44^. In an endotoxin-induced mice BPD model, exogenous recombinant ANG-1 reported to inhibit alveolar simplification^45^. In regard to the relation with SPMs, a recent study using the mice hindlimb ischemia model showed that exogenous RvD1 up-regulated pro-revascularization genes such as ANG-1 and VEGF receptor (VEGFR) to promote vascular remodeling, and enhanced perfusion recovery^46^. These results are consistent with ours, showing a favorable effect of RvD1 on vascular growth, specifically on the capillaries < 20 µm in diameter, after a Bleo-induced lung injury.

Among the five genes identified to explore the genetic background of the effects of RvD1, all but ADAMTS-12 were significantly suppressed in their upregulation by Bleo. IGF-1 Receptor (IGF-1R) knockout mice are lethal, displaying severe lung hypoplasia (less than a quarter of normal size) with arrested lung development at the canalicular stage^42^. A phase 2 trial of recombinant human (rh)IGF-1/ IGF-binding protein (IGFBP)-3 complex (Mecasermin rinfabate) reporting its efficacy in preventing the development of the most severe form of BPD^47^. These studies clearly indicate that IGF-1 is essential for lung development and repair, and has an inhibitory effect on BPD development. However, immunostaining performed in the autopsy cases of BPD revealed increased levels of IGF-1 and IGF-1R^48^. Also, in the hyperoxia-induced rat BPD model, IGF-1, IGF-1R, and IGFBP are all upregulated^49^. During normal lung development in mice, IGF-1 expression peaks around PD-3 (early alveolarization stage) and then declines^43^. According to an animal model of intrauterine growth restriction induced by maternal nutritional restriction (also known as another BPD model), IGF-1 expression is decreased, and intrinsic-pulmonary IGF-I signaling is suppressed during the fetal and early neonatal periods; however, during the later catch-up growth phase, both IGF-I and IGF-I signaling are elevated, and septal thickness, matrix deposition, and increased expression of vimentin and collagen Iα1 are observed due to the activation of myofibroblasts^50^. In summary, IGF-1 is essential during alveolarization phase, but in secondary septation phase after PD-14, during which it should decrease, IGF-1 may instead inhibit alveolar development. Therefore, the suppression of IGF-1 expression by RvD1 at PD-14 may have a beneficial effect.

TNC is a glycoprotein, one of the components of extracellular matrix (ECM), and transiently expressed during organogenesis in fetal to early postnatal period. It virtually disappears in adult maturation tissues, whereas reappears temporarily in pathological conditions such as inflammation and tumorigenesis^51^. During normal murine lung development, TNC expression shows a biphasic peak; during the embryonic airway branching phase and during early alveolarization phase after birth (peak around PD-7) and decreases again on PD-14^51^. TNC knockout mice show a normal phenotype with impaired alveolarization and microvascular maturation only during postnatal period, which later becomes normalized in adulthood^51^. TNC expression is elevated in hyperoxic murine BPD models^16^ as well as in infants with BPD^52^, when it should ideally reduce and disappear. Bao et al. performed a long non-coding RNA screen in a hyperoxic mice BPD model, using PD-7 lung tissue, and found that reduced expression of a long non-coding RNA AK033210, which associates with TNC expression, may be part of the pathogenesis of BPD^53^. Recently, Liu et al. subjected lung tissue from normal mice during the alveolar differentiation stage to perform proteomic analysis and then further filtered the results using bioinformatic methods to identify TNC as one of the ECM protein candidates most likely to be associated with the development of BPD^54^. Using both a hyperoxic BPD model and in vitro studies, they found that TNC exhibited a dual role in lung alveolarization depending on its concentration: low-dose TNC promoted proliferation and migration of lung epithelial cells, while high-dose TNC had the exact opposite effect^54^. Taken together, suppression of increased TNC expression by RvD1 may be part of the molecular basis for its inhibitory effect on Bleo-induced BPD-like alveolar dysmorphogenesis.

Quiet interestingly, Olave et al. selected microRNA-489, which targets both IGF-1 and TNC, as a candidate biological regulator involved in alveolar septation according to unbiased microRNA transcriptomic analysis using developing mice lung tissue^55^. Hyperoxic exposure increased the expression of both IGF-1 and TNC at PD-14 (end of alveolarization or start of secondary septation), but unexpectedly, knockdown of microRNA-489 accompanied by a further increase in both IGF-1 and TNC resulted in amelioration of the hyperoxia-induced alveolar dysplasia, while overexpression of microRNA-489 inducing suppression of the increase in both IGF-1 and TNC led to the opposite result^55^. This result reconfirms that both IGF-1 and TNC are more than just more or less, but that it is essential to maintain a delicate quantitative balance depending on the stage of lung development.

ELN is a major component of connective tissue together with collagen. ELN knockout mice are postnatal lethal and their lungs show arrested terminal airway branching with fewer distal air sacs^56^. During normal murine lung development, ELN expression peaks around PD-4 to 14 (alveolarization phase)^57^. In the hyperoxia-induced murine BPD model, ELN expression is increased in the alveolarization phase after PD-14, and treatment with lipoxin A_4_^16^ or mesenchymal stem cell^58^ suppresses its enhanced expression during this period, along with normalization of alveolar structure. It is well known that the lungs from the infants with BPD shows an increase in elastic tissue with perturbated appearance^59^. Therefore, the suppression of enhanced ELN expression in PD-14 by RvD1 in our study may have also helped to improve alveolar simplification.

ANLN is not only essential for cytokinesis, but is known to be overexpressed in various solid tumors and is closely linked to their progression and metastasis, leading to poor prognosis^60^. It has been suggested that one of its molecular mechanisms promoting tumor metastasis may be related to the epithelial-mesenchymal transition^61^. This phenomenon is also seen in BPD models, where the alveolar epithelium is transformed into myofibroblasts, promoting fibrosis^62^. Therefore, some of the effect of RvD1 in our model may be via suppression of ANLN; however, α-SMA staining failed to reveal any significant findings (Fig. 4).

This study had several limitations. First, the administration of RvD1 was initiated at the same time as that of Bleo, which is clinically equivalent to prophylactic use. Therapeutic use of RvD1 should be possible, even if the initial inflammation is uncontrolled. However, its efficacy, when administered in the later phases of inflammation, must be studied further. Second, the examination was not performed during the period preceding d-14, thus rendering it uncertain whether RvD1 truly has no effect on the acute phase of inflammation. Nevertheless, there is a wealth of literature investigating the mechanisms of action of SPMs, including RvD1, and these findings are widely accepted^11,14,20^. It is, therefore, plausible that this study may be similarly confirmed. Third, changes in selected gene expression at the protein level were not confirmed using western blotting. Fourth, studies are needed to verify whether cytokine switches (inhibiting pro-inflammatory cytokines/chemokines and promoting pro-resolving, anti-inflammatory cytokines/chemokines, as shown in Supplementary Fig. S1 online), occur in this model; however, the samples available for this analysis were insufficient. Additionally, Bleo administration is common as an IPF model but not popular as a BPD model^63^. In principle, it is impossible to mimic the complex pathologies of BPD in any model other than preterm Catarrhine monkeys^64,65^. At the very least, the Bleo-newborn murine model is one of the better models that faithfully reproduces some aspects of the pathology of BPD^22–26^.

In conclusion, RvD1 improved alveolar structural changes, reduced macrophage infiltration and promoted the repair of capillary damage in the Bleo-induced model of alveolar maturation arrest of BPD. The molecular basis of the RvD1 effect appeared to be the prevention of overexpression of IGF-1, TNC, ELN, and ANLN genes by Bleo. SPMs are ideal biological substances that facilitate only the resolution of inflammation without interfering in its essential role, such as the elimination of foreign substances and damaged tissue. Clinical trials have already started in various chronic inflammatory diseases^20^. Presently, the final report on the clinical trials involving resolvin preparations is not available, leaving us to consider the potential adverse effects only speculatively. However, it is conceivable, in theory, that if a new inflammatory mechanism is initiated during administration of resolvin, it could possibly interfere with the inflammatory phase and potentially affect the fundamental purpose of inflammation. It will not be long before SPMs, including RvD1, appear in clinical practice as promising therapeutic agents for BPD.

## Methods

### Ethical approval

The experimental protocol, including the ethical aspects, was approved by the Osaka Medical and Pharmaceutical University Animal Care and Use Committee (authorized number 25045, 26013, and 27041). All experiments were performed in accordance with their guidelines and regulations. This study is reported in accordance with the ARRIVE guidelines.

### Animals

Specific-pathogen-free, timed-pregnant Sprague–Dawley rats were purchased from CREA Japan, Inc. (Tokyo, Japan) at 18 days of gestation. Spontaneously delivered newborn pups at 21 days of gestation were housed with dams and fed ad libitum under a 12-h light–dark cycle. The day of birth was defined as PD-0.

### Chemicals

RvD1 was purchased from the Cayman Chemical Company (Ann Arbor, MI), and bleomycin sulfate was obtained from LKT Laboratories, Inc. (St. Paul, MN). 2-hydroxypropyl-β-cyclodextrin (HBC) for dissolving RvD1 was purchased from Sigma-Aldrich (St Louis, MO). Anti-CD68, anti-CD31 and anti-α-SMA antibodies were obtained from Abcam, Inc. (Cambridge, UK), anti-CD86, anti-CD163, and anti-IGF-1 was from Bioss, Inc. (Woburn, MA), and anti-Ly-6G antibody was from eBioscience, Inc. (San Diego, CA).

### Experimental protocol

**Fig. 6:**
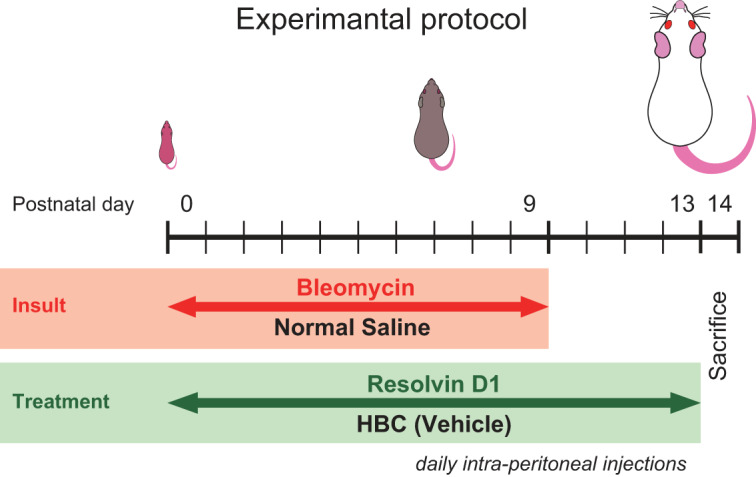
Study protocol. Bleo (1 mg/kg of body weight) or normal saline was injected intra-peritoneally from PD-0 to PD-10. RvD1 (500 ng), or vehicle (2-hydroxypropyl-β-cyclodextrin, HBC) was injected until PD-13. The pups were sacrificed on PD-14.

Immediately after birth, the pups were randomly divided into four groups: *Control* (0.9% normal saline + HBC), *Bleo* (Bleo in normal saline + HBC), *Bleo* + *RvD1* (Bleo in normal saline + RvD1 in HBC), and *RvD1 alone* (0.9% normal saline + RvD1 in HBC). Bleo in normal saline or normal saline alone were injected for the first 10 days (PD 0–9). One hour after these injections, RvD1 in HBC or HBC alone was administered throughout the study period (PD 0–13). On the PD-14, the pups were euthanized with a lethal dose of phenobarbital (Fig. 6).

To develop a BPD-like arrested alveolarization model, Bleo was administered at a dose of 1 mg/kg of body weight^22–24^. As regards the dosage of RvD1, previous animal studies reported a range of 20–400 ng (based on the assumption that our rat pups weighed 25 g and adult mice weighed 19 g)^66–72^. Therefore, we initially experimented with a single dose of 50 ng of RvD1 injected on PD-0; however, no morphological difference was observed between the Bleo and Bleo + RvD1 groups. A single dose of 500 ng was also ineffective. Based on these results, we switched to daily dosing and first tried 50 ng daily, which was still ineffective. Finally, we observed treatment efficacy when the dose was increased to 500 ng. Accordingly, we proceeded with the 500-ng dosage daily for 10 days. All reagents at a volume of 5 µL were injected intra-peritoneally by a 26-gauge needle.

### Tissue preparation

After the rats were euthanized, the trachea was exposed, the airway was secured by tracheostomy, and artificial respiration management was initiated. The lungs and heart were exposed by thoracostomy, and a small incision was made in the right atrium to remove the blood, after which the right ventricle was punctured. PBS was infused into the main pulmonary artery through the right ventricular cannula to flush blood from the pulmonary circulation. The left main bronchus and pulmonary artery were ligated, and the left lung was dissected. The left lung was snap-frozen in liquid nitrogen for subsequent RNA and protein analysis and stored at − 80℃. After removing the left lung, the right lung was inflated at 25 cmH_2_O pressure with 4% paraformaldehyde in PBS for 5 min. They were then closed with a suture, and the lungs were fixed in buffered formaldehyde for 24 h at 4 °C. Paraffin-embedded sections of formalin-fixed lung tissues were stained with hematoxylin and eosin (H&E).

### Lung morphometric analysis

H&E-stained sections were photographed from at least 10 random locations in a single lung using a Nikon Eclipse 80i microscope at 10 × magnification and captured with a digital camera (DS-Ri1, Nikon, Tokyo, Japan). The measurements were performed in three non-consecutive sections by a single observer blinded to the group identity.

Alveolarization was assessed by measuring RAC using the method described by Emery and Mithal^73^. From the center of the respiratory bronchiole, the shortest line was drawn to the edge of the acinus, and the number of septa crossed by the line was counted.

The intra-alveolar distance was measured as MLI using the established standard methods^74^. MLI was determined by dividing the total length of the 42 lines drawn across the lung section by the number of intercepts observed.

The mean ST was calculated using the images photographed at 200 × magnification under a grid of five equally spaced horizontal lines. The thickness of each septum crossing a given horizontal line was measured perpendicular to its course at the crossing point^75^. Ten separate grids were analyzed, and the average ST was then calculated for each grid.

### Immunohistochemical analysis

Paraffin-embedded Sects. (4 μm) of formalin-fixed right lung tissue were prepared for immunohistochemical analysis. Serial dehydration was sequentially performed by immersing them in 100% ethanol, 90% ethanol, 70% ethanol, and lastly, in water. After heating in the microwave, they were retrieved by washing with PBS. Endogenous peroxidase activity was reduced by immersion in 3% hydrogen peroxide. After rinsing, the sections were immersed in 3% goat serum for 30 min and incubated with 25 μg/mL murine-derived anti-IGF-1, anti-CD31, anti-CD68, anti-CD86, anti-CD163, anti-Ly-6G, or anti-α-SMA antibodies diluted in PBS (1:50,1:500,1:400,1:100, 1:100, 1:200, or 1:6400, respectively) overnight. Thereafter, the sections were incubated with a biotin-labeled secondary antibody (1:300) for 2 h and subsequently incubated with an avidin–biotin complex staining kit (Vector Laboratories, Burlingame, CA) for 30 min at approximately 25 °C. They were then subjected to development with diaminobenzidine and hydrogen peroxide. Slides were lightly counterstained with hematoxylin and dehydrated by sequential immersion in 70% ethanol, 90% ethanol, 100% ethanol, and 100% xylene before applying the coverslips. Each section was visualized using a Nikon Eclipse 80i microscope with a 40 × objective.

For immunohistochemical analysis of other than IGF-1, the positively stained cells per high-power field (100 × magnification) were counted. For analysis of IGF-1, we first evaluated the staining intensity on a five-point scale (point 1: almost not stained to point 5: extremely stained) for bronchial epithelium, alveolar epithelium, endothelial epithelium, and smooth muscles, and then counted the number of positively stained cells in 0.01 mm^2^ × 30 slices for each part of the epithelium, expressed as a percentage. Finally, the staining index (staining intensity × percentage of stained cells) was calculated as previously described^48^.

### Real-time PCR

A part of the frozen lung tissue was used to investigate the gene expression. Total RNA was extracted using ISOGEN (Nippon Gene, Tokyo, Japan) according to the manufacturer’s protocol. The RNA purity was verified using the A260/A280 ratio. Total RNA was reverse-transcribed into cDNA using the Omniscript Reverse Transcription Kit (Qiagen, Venlo, Netherlands). The cDNA was then amplified by real-time quantitative TaqMan PCR using TaqMan Gene Expression Master Mix (Applied Biosystems, Life Technologies, Carlsbad, CA) and a StepOnePlus real-time PCR system (Thermo Fisher Scientific, Waltham, MA), using a modified version of the manufacturer’s protocol. Glyceraldehyde-3-phosphate dehydrogenase (GAPDH) was used as a standard. The expression level of each mRNA was calculated using the 2^-ΔΔCT^ method. The following inventoried TaqMan primers (TaqMan Gene Expression Assays) were used in this study: ANLN (Rn01749935_g1); PLA2G2A (Rn00580999_m1); TNC (Rn01454948_m1); ELN (Rn01499782_m1); IGF-1 (Rn00710306_m1); ADAMTS-12 (Rn04418968_m1).

### Statistical analysis

Data are expressed as mean ± SD. The differences between the groups were assessed by one-way ANOVA with a post-hoc Tukey test using BellCurve for Excel (Social Survey Research Information Co., Ltd., Tokyo, Japan). A *p*-value < 0.05 was considered statistically significant. The size of the effect was estimated from the distribution by calculation of Cohen’s *d*.

## Supplementary Information

Supplementary Information.

## Data Availability

All data generated or analyzed during this study are included in this article and its online supplementary material files. Further inquiries can be directed to the corresponding author.

## Abbreviations

ADAMTS-12: A disintegrin and metalloprotease with thrombospondin domains
α-SMA: α-Smooth muscle actin
ANG-1: Angiopoietin-1
ANLN: Anillin actin-binding protein
Bleo: Bleomycin
BPD: Bronchopulmonary dysplasia
ECM: Extracellular matrix
ELN: Elastin
H&E: Hematoxylin and eosin
HBC: 2-Hydroxypropyl-β-cyclodextrin
IGF-1: Insulin-like growth factor-1
IGFBP: IGF-binding protei
IPF: Idiopathic pulmonary fibrosis
MLI: Mean linear intercept
NS: Not significant
PD: Postnatal day
PLA2G2A: Phospholipase A2 group IIA
RAC: Radial alveolar count
RvD1: Resolvin D1
SPM: Specialized pro-resolving mediator
ST: Septal thickness
TNC: Tenascin C
VEGF: Vascular endothelial growth factor
VEGFR: VEGF receptor
