# Supplementary material for: Resolvin D1 improves bleomycin-induced alveolar maturation arrest in newborn rats
Source: Sci Rep. 2025 Aug 5;15:28554. doi: 10.1038/s41598-025-12739-4 (PMC12325681; doi:10.1038/s41598-025-12739-4)
Supplement: Supplementary file 1 — Supplementary Information. [file 41598_2025_12739_MOESM1_ESM.pdf]

Supplementary Figure S1 Biosynthesis and pro-resolving anti-inflammatory effects of RvD1

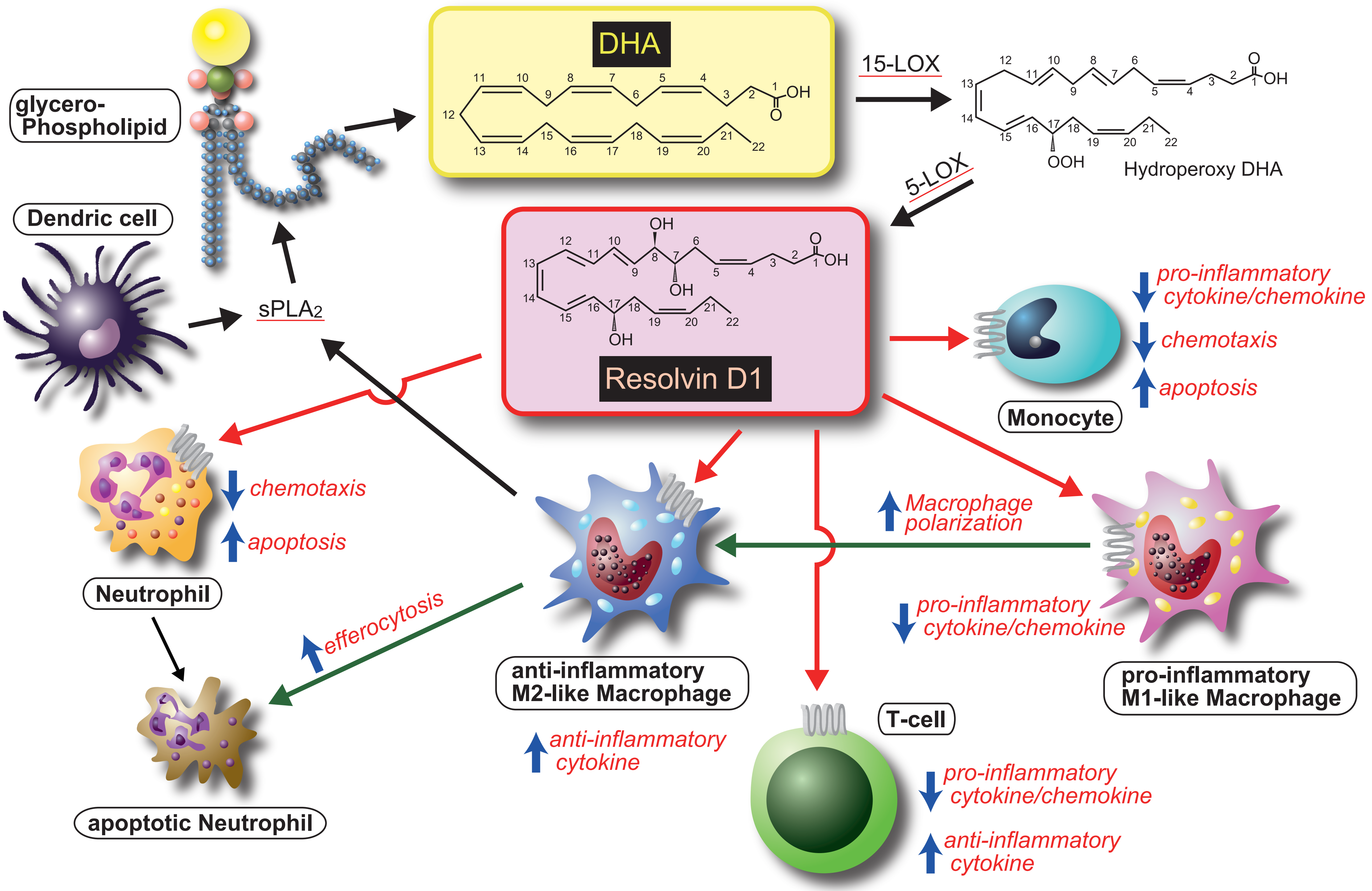

Supplemental Figure S1: Biosynthesis and pro-resolving anti-inflammatory effects of RvD1

DHA, the mother molecule of RvD1, is mainly present as a component of PE series glycero-phospholipid. The phospholipases with high affinity for ω3-PUFA are reported to be sPLA2-IID, -IIF and -X. sPLA2-IID is mainly expressed in dendritic cells as well as M2-like phenotype macrophages, and hydrolyzes the sn-2 position of PE in various microparticles to release DHA. DHA is firstly converted into 17S-hydroperoxy-DHA by endothelial 15-LOX, and RvD1 is sequentially produced by leukocyte 5-LOX through an epoxy intermediate. RvD1 binds to the 7-transmembrane G-coupled receptors, formyl peptide receptor 2 (ALX/FPR2) or human RvD1 receptor (DRV1/GPR32), and exerts its action. RvD1 regulates the overall production of cytokines/chemokines by acting on monocytes, macrophages, T-cells, etc.: inhibiting pro-inflammatory and promoting pro-resolving, anti-inflammatory ones. It limits chemotaxis and induces apoptosis of neutrophils and monocytes. It promotes polarization of M1-like macrophages to M2-like, pro-resolving phenotype, and for M2-like macrophages, enhances clearance of debris and apoptotic neutrophils (efferocytosis). Overall, RvD1 acts in the direction of resolving active inflammation and restoring tissue damage.

DHA, docosahexaenoic acid; RvD1, resolvin D1; PE, phosphatidylethanolamine; PUFA, polyunsaturated fatty acid; sPLA2, secreted phospholipase A2; LOX, lipoxygenase; VEGF, vascular endothelial growth factor.

Supplementary Figure S2

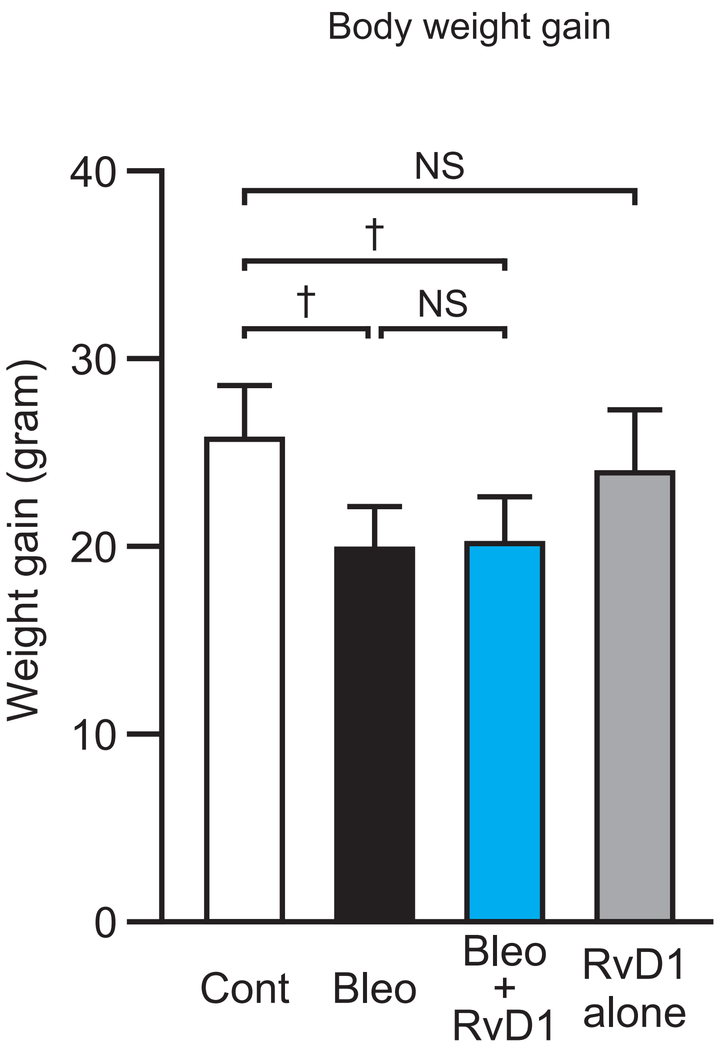

Supplemental Figure S2: Body Weight

Mean weight gain from birth to PD-14 (the end of the experiment). White bar, Control group; black bar, Bleo group; blue bar, Bleo+RvD1 group; gray bar, RvD1 alone group. Mean ± SD. N = 5–8/group. † p<0.001, NS: not significant. Bleo caused weight loss, and RvD1 was unable to halt this loss. However, RvD1 itself did not influence body weight.

Supplementary Table-S1

List of genes with considerable changes in patients with BPD/IPF, bleomycin models, and mammalian lung embryogenesis

	Human BPD[37]	Bleomycin model[35,36]			Common to Bleo model and IPF[36,38]	Lung development Mouse[39]	Lung development Human[39,40]			
		Inflammatory response	Fibrotic response	Resolution						
	acp5	Adm	Adam8	Akrlc21	aadac	Acml4	ABI3BP		RED	common to Bleo, BPD, and IPF
	adamts16	Aocs3	Adam9	Arid3b	adam12	Adam12	APOA2		GREEN	common to Bleo and BPD
	adcyap1	Aps	Adcy2	ASPA	adamts12	Adam3	AQP4		BLUE line	common to Bleo, mouse lung development, and IPF
	adcyp1r1	Atp1a3	Add1	Atp6v1b1	adamts4	Adamts12	ASPA		***BLUE line italic bold***	common to Bleo, human lung development, and IPF
	adora3	Ax1	Adprh	Brp17	Adrb2	Adra1a	ATP11A		BLUE	common to Bleo and mouse lung development
	ager	B3gnt7	Aldoa	Capza1	Afap1l1	Aif1l	ATP8A1		PURPLE	common to BPD and mouse lung development
	aldh1a3	Bax	Anln	Cdc14b	ankrd34b	Aim1	B3GALT2		PURPLE line	common to BPD and human lung development
	alpp	C3ar1	Arf2	Cdon	anln	Alc9a4	BCL6			
	anln	Camk1	Arf6	Clec2	Anxa3	Aldh1a1	BCMP11			
	anxa8l2	Carf	Arhgap1	Cngb3	asf1b	Ampd3	C10orf79			
	apobec3b	Ccr1	Arl2	Cpne6	aspm	Angpt2	C13orf30			
	apoc2	Cd33	Arl6ip5	Crmp1	atg9b	Aox3	C19orf33			
	apoh	Cd68	Arnt	Dffb	atp6v0d2	Apol8	C1orf192			
	arntl2	Cdkn1a	Arpc1b	Dhx16	aurka	Arhgap28	C1orf88			
	aspm	Chl1	Atp6v1b2	Ebag9	aurkb	Armcx2	C20orf85			
	atp6v0d2	Ckb	Bag2	Ecgf1	bcat1	auts2	C9orf135			
	bhlhe22	Clecsf10	Bcl10	Egln3	bhlhe22	B2 m	C9orf24			
	birc5	Clecsf6	Bgn	Fabp4	Bik	BC006779	CAPSL			
	bub1	Cmkor1	Bin1	Fabp4	bub1	Bcl6	CCND2			
	c10orf10	Col5a1	Cald1	Fbxo22	Bub1b	Blnk	CD44			
	c1qa	Col5a3	Car13	Fkhr1	c12orf48	Brd3	CD74			
	c1qb	Coll2a1	Cast	G0s2	c12orf53	Btnl9	CDC25A			
	c20orf114	Colla1	Ccnd1	Ggtl3	c12orf75	Camk1 g	CEBPD			
	c6orf105	Colla2	Ccnd3	Gpr3	c15orf48	Ccdc36	***CENPN***			
	c7orf68	Coro 1c	Cd53	Gpr49	c1orf135	Ccl5	CLDN18			
	ca12	Cotl1	cdc42se1	Gria3	c1qb	Ccnjl	CLIC3			
	ca4	Cpz	Cdc42sp5	Grik5	c1qc	Ccr6	CLIC5			
	ccdc102b	Csflr	Cdk2	Gsh1	c1qtnf3	Cd109	CTNND2			
	ccl18	Ctsb	Chst4	Harsl	c1qtnf6	Cd19	CXCL5			
	ccl22	Ctsd	Clca4	Hpgd	c2orf40	Cd209a	CYorf15B			
	cd177	cyb561	Col4a2	Ikbkb	c3ar1	Cdh11	DEPDC1			
	cd5l	D15Wsu75e	Commd10	Ing1l	C6	Cdhr3	DNHD2			
	cd8a	D2Wsu81e	Cpxm2	Kcns1	Calca	Cdk5rap2	DYNLRB2			
	cdc25c	Dlst	Cxcl4	Khdrbs1	capn6	Cenpn	ELL2			
	cdcp1	Dpysl3	Cyba	Lgals6	casc5	Chi3l3	FAM81B			
	cdk1	Dscr1	Ddit4	Mepe	Cav1	Chn1	FLJ23049			
	cdkn3	Edg5	Dnaja4	Metapl1	Cav2	Cit	FLJ44379			
	ceacam5	Edr23	Doc1	Mybl1	Ccdc68	Ckap4	FOLR1			
	ceacam6	Eps8l1	Dsr3	Narg2	ccdc80	Cobl	FSTL5			
	cenpa	Erp29	E2f1	Nr2e3	ccdc99	Col22a1	FXBP5			
	cga	Fcer1g	Edg1	Nras	ccna2	Col27+B5:H75a1	GLDC			
	ch25h	Fcgr3	Eef1g	Nudt13	Ccnb2	Col6a3	GRHL1			
	chit1	Fes	Egln1	Olfr491	ccne1	Crebl2	GTSE1			
	col4a6	Fmod	Evl	Pax3	ccnf	Ctnna2	HLA-DPA1			
	col8a1	Fst	Ewsr1	Phtf1	ccng1	D43004 1D05Ri k	HLA-DRA			
	comp	Fut7	Fscn1	Poli	ccr5	Dbc1	HLA-DRB1			
	cpa3	Gabpb2	Fuca2	Ppp1r1b	cd276	Dctd	HMGA2			
	csmd1	Galc	G6pc3	Ppp2r5a	cdc20	Dennd2a	IFI27			
	cxcl13	Galns	Gas6	Prlpc2	cdc25c	Dgki	INMT			
	CXCL5	Gdfl5	Gmpr	Pus3	cdca2	Dido1	INSM1			
	cxcl5	Gjb3	Gpc1	Rabep2	cdca3	Dlk1	KCNJ15			
	cyp24a1	Gjb5	Gpx7	Rbed1	cdca5	E2f7	KCNJ5			
	depdc1	Gp49a	Gsr	Rps7	cdca8	E2f8	L1TD1			
	dhrs9	Gusb	Gtf2f1	Scnl1a	cdk1	Efemp1	LAMP3			
	diaph3	Gyltl1b	Gys3	Slc19a1	cdkn3	Egln3	LOC493869			
	dio2	Hdn	Hemp1	Star	cenpe	Eln	LPAAT-t			
	dkk1	Hexa	Hexb	Surf6	cenpf	Eme1	LPAAT-THETA			
	dnajc5b	Hrmt1l2	Ier5	T2	cenph	Ermap	MALL			
	dok5	Hsd1b2	Ifnar2	Taz	cenpk	Ext1	MCL1			
	edn3	Itgb3	Islr	Usp29	cenpm	Fancd2	MFSD2			
	ereg	jak3	Isynal1	V1rc7	cenpn	Fanci	MGC42367			
	fabp4	Kcne4	Itm2c	Xpa	***CENPN***	Fat4	MICAL2			
	Fabp6	Lfi30	Itsn1	Zfp143	cenpp	Fbln5	***MME***			
	fcer1a	Lgmn	Kdelr3	Zfp278	cep55	Fbn2	MUC1			
	ghrl	Igsf7	Lepre1	Zfp386	cfi	Fech	ODAM			
	gpihbp1	Lhfpl2	Lztr1		cgref1	Flt3	***OLFML2A***			
	gpr109b	Lox12	Man1b		ch25h	Frem2	PIK3C2G			
	grem1	Lrp10	Manba		chek1	Gbp5	RANBP9			
	gstt1	Lyl1	Mapk3		chl1	Ggct	RASGRF1			
	hdc	Lypla3	Mapk6		chsy3	Glb1l3	RFXDC1			
	hhip	Map17	Mark2		ckap2l	Gli3	SDC4			
	hla-drb4	Mapk13	Mbc2		clec5a	Gm281	SFRP2			
	hs3st2	Mclr	Mknk1		Clic5	Gm6531	SFRP2			
	igf1	Mfge8	Mlc1		clip	Grap2	SFTPB			
	igha1	Mkiras2	Mpp1		clspn	Grem2	SFTPC			
	ighm	Mmp12	Myd88		col15a1	Gria1	SFTPD			
	igj	Mmp8	Myo10		Col1a2	Gypa	SLC16A3			
	igkc	Mrg2	Myo1c		col24a1	H2-Ob	SLC34A2			
	il17rb	Ms4a11	Nde1		col3a1	Heatr1	SLC35D3			
	il1rl1	Ms4a6d	Neo1		col5a1	Hs6st3	SMPX			
	inhba	Msn	Nfkb2		col5a2	Id4	SPAG6			
	iqgap3	Msr2	Nfya		col8a1	Ifit3	SPATA18			
	itln1	Mtvr2	Odf2		cps1	Ifngr1	ST6GALNAC5			
	kank4	Myadm	Osbpl5		cpxm1	Igfbp3	STAMBPL1			
	kcnj16	Ncf1	Ostf1		crabp1	Igsf10	STEAP4			
	kiaa0101	Nos2	Pgm1		crh	Il6st	STMN2			
	kif14	Nrg3	Pkp3		cthrc1	Inmt	STX7			
	kif18b	Olfr127	Pmm1		Ctse	Irak1bp	SULF1			
	kif23	Olfr1360	Pofut2		ctsk	Kcnj15	***TMEM100***			
	kmo	Olfr502	Pros1		cxcl12	Kcnma1	TSGA2			
	krt17	Olr1	Ptk9		cxcl13	Kif2c	***UCHL1***			
	lgals3	Otub1	Ptpn1		cyp7b1	Klb1b	***VEPH1***			
	lif	Pip5k2c	Pygb		darc	Lama1				

lipg	Pkm2	Rbm28	dbn1	Lhfpl2
lppr1	Plat	Rcn3	dclk1	Lilrb3
lrrc15	Pld3	Rgs19ip1	ddit4l	Lims2
lrrc2	Plod3	Rhoc	depdc1	Lmnb1
marco	Polr3d	Rhog	dlgap5	Lrg1
mki67	Pop7	Rtn4	Dnase2b	Lrp2
mnd1	Prad	Scmh1	dscc1	Lrr1
ms4a2	Prrx2	Scotin	e2f7	Lrrm3
msmb	Psap	Sec14l1	e2f8	Lrrn1
mxra5	Ptpns1	Sec5l1	eln	Ly6a
neil3	Pxn	Sept(in)8	Epb41l5	Ly6c1
nek2	Rab3il1	Sesn2	esco2	Maged1
nnmt	ralb	Sgpp1	Esm1	Maged2
olfm3	rap2ip	Siat5	espl1	Mcm3
p4ha3	Rhbdl6	Slc35a4	exo1	Mex3a
pappa	Rnf36	Slc37a2	f7	Mfap4
pbk	Samd8	Sod3	fam3b	Mmp3
pcolce2	Scap3	Spg7	Fap	Mtmr7
Pla2g2a	Scn1b	Srf	fbn1	Ncam1
plunc	Serpinb9	Steap	fbn2	Nid2
posten	Sertad1	Stx6	fcgr2a	Nrm1
pou2af1	Sfrp1	Surf5	fcgr2b	Oas2
prnd	Slc10a3	Tcfe3	Fgg	Obsl1
psca	Slc12a4	Tes	Fhl2	Pa2g4
psph	Slc15a3	Tesk1	fignl1	Pank1
rbp4	Slc22a	Tfdp1	Fkbp10	Pdlim3
rorc	Slc7a1	Tm4sf15	Fkbp11	Pfdn1
rrm2	Slc7a7	Tm4sf9	fmod	Phf15
rtkn2	Smr2	Tmem2	fn1	Pitpnc1
s100a3	Sorcs2	Tpm3	foxm1	Plac1
sele	Sp5	Traf5	fst	Ppp2r5c
serpina1	Sphk1	Tuba1	Gal	Prdm8
sfn	Spred2	Tubb5	gas2l3	Prdx4
sfrp2	Sprr1a	Ubtd1	gdf15	Prdx6
sh3rf2	Stac2	Uxs1	gins2	Prkcq
siglec1	Stk22a	Vapb	gjb3	Ptpla
siglec6	Syngr1	Vcl	gjb5	Ptx3
ska1	tacstd2	Wwtrl	Gpd1	Rasgrp1
slc18a2	Tbxas1		gpnmb	reat1e
slc27a6	Tgfbi		grem1	Rfc5
slc44a5	Tgfbr2		gtse1	Rhou
slco1a2	Tgm2		hmmr	Rnf213
steap1	Thbs6		Hpgd	Robo1
tart	timp1		ibsp	Rrm1
tdo2	Tm7sf1		igf1	Rrm2
themis	Tmp		igj	Scd1
tlgap5	Tnc		il12b	Scml2
tmem158	Tnfrsf23		il1rn	Scml4
tmtc1	Tnfrsf2a		il2ra	Scube2
tnip3	Trem2		itgax	Serping1
top2a	Trim35		itgb6	Sh2d1a
tpsab1	Trpv3		Kcnn4	Sh3bp5
tpsb2	Tsc36		kiaa0101	Shh
tpx2	tslpr		kiaa1199	Shisa2
ttk	Ttyh2		kiaa1524	Shisa3
ttll6	Ubadc1		kif11	Shisa6
ugt4a6	Ube2e2		kif18b	Ska1
upk3b	Unc119		kif20a	Skp2
vgll1	Unc93b1		kif22	Slc27a6
xist	V2R2		kif23	Slco3a1
	Vhlh		kif2c	Snca
	Xpnpep1		klhdc8a	Sod3
			kntc1	Sox9
			kynu	Sp110
			lair1	Sp140
			Lcn2	Spag5
			lhfpl2	Spnb5
			Lifr	St3gal1
			LOC691221/C5orf4	Stat4
			lrrc15	Stfa1
			Lrrn3	Tcf21
			lum	Thrap3
			mafb	Tiam2
			mark1	Tmcc3
			mastl	Tmem28
			megf10	Tmsb10
			melk	Tnc
			mex3a	Ttf2
			MGC105649/C15orf48	Tubb5
			Mia	Unc5c
			Mlf1ip	Wdfy4
			Mme	Wif1
			MME	Wisp2
			mmo10	Zbtb16
			mmp13	Zbtb4
			mmp14	Zdhhc2
			mmp2	Zfp618
			Mmp7	
			Mnd1	
			mthfd2	
			Mycn	
			myo5a	
			nav3	
			ncapg	
			ncapg2	
			Nebl	
			Nedd9	
			neil3	
			nek6	
			nnat	
			Npr3	
			Nuf2	
			nusap1	

					Olfml2a					
					OLFML2A					
					p4ha3					
					pappa2					
					Pard6b					
					pbk					
					pdgfc					
					Phactr1					
					phek					
					pi15					
					pif1					
					pkp1					
					Pla2g2a					
					Pla2g7					
					plcd4					
					Pln					
					pmch					
					Podnl1					
					Prkce					
					prox1					
					prrx2					
					prune2					
					psrc1					
					ptger3					
					Ptges					
					Ptprr					
					rad51					
					rad51ap1					
					Radil					
					Ramp2					
					Rapgef5					
					RGD1306437/C13orf1					
					RGD1311307/C6orf145					
					rgs1					
					rhbdl2					
					S1pr5					
					saa1					
					scamp5					
					Sdpr					
					Sel1l3					
					Selenbp1					
					serpina10					
					sfrp1					
					Sgef/ARHGEF26					
					sgol1					
					Sh3bp5					
					sh3pxd2b					
					shc4					
					shcbp1					
					siglec1					
					slamf9					
					Slc1a1					
					slc26a4					
					Slc2a5					
					slc37a2					
					slc39a14					
					Slc4a1					
					slc7a11					
					slfn13					
					smc2					
					spc25					
					spink1					
					spp1					
					stac2					
					stil					
					Stxbp6					
					syt12					
					Tal1					
					tfap2a					
					tfec					
					Thbd					
					thbs1					
					thbs2					
					thbs4					
					timp1					
					tk1					
					TMEM100					
					Tmem213					
					tnfaip8l1					
					tnfsf11					
					Top2a					
					tpx2					
					trem2					
					trip13					
					Tspan12					
					ttk					
					tub					
					tubb3					
					ubd					
					ube2c					
					ube2t					
					UCHL1					
					uhrf1					
					vcan					
					Vegfa					
					VEPH1					
					vsig4					
					wisp1					
					ync					
					Zfp365					
					zranb3					
					zwilch					

Supplementary Table-S2

Selected list of genes that seemed most relevant to our Bleo model

Final selection	Second selection	First selection	description
		adam12	cell-cell and cell-matrix interactions, including fertilization, muscle development, and neurogenesis.
adamts12	adamts12	adamts12	Zinc metalloproteonase with thrombospondin motif. Realated to placenta formation, arthritis, modulationand recovery of infllamatory process.
anln	anln	anln	a role in cell growth and migration, and in cytokinesis.
		ASPA	enzyme that catalyzes the conversion of N-acetyl_L-aspartic acid (NAA) to aspartate and acetate
		aspm	mitotic spindle regulation, with a preferential role in regulating neurogenesis
		atp6v0d2	proton transmembrane transporter activity
		bhlhe22	cerebral cortex regionalization
		bub1	serine/threonine-protein kinase that play a central role in mitosis.
		c1qb	B-chain polypeptide of serum complement subcomponent C1q
		cdc25c	regulation of cell division
		cdk1	essential for G1/S and G2/M phase transitions of eukaryotic cell cycle.
		cdkn3	cyclin-dependent kinase inhibitor, and has been shown to interact with, and dephosphorylate CDK2 kinase,
		CENPN	bound to kinetochores during S phase and G2 and recruits other proteins to the centromere
		ch25h	cholesterol 25-hydroxylase activity. Involved in B cell chemotaxis
	col8a1	col8a1	one of the two alpha chains of type VIII collagen. basement membrane of the corneal endothelium. Realated to cancer progression.
		cxcl13	B lymphocyte chemoattractant,
		CXCL5	Chemokines, which recruit and activate leukocytes
		depdc1	GTPase activator activity. Involved in negative regulation of transcription
		e2f7	essential role in the regulation of cell cycle progression
		e2f8	progression from G1 to S phase by ensuring the nucleus divides at the proper time
		Egln3	activation of cysteine-type endopeptidase activity involved in apoptotic process and response to hypoxia
Eln	Eln	Eln	one of the two components of elastic fibers
	fabp4	fabp4	fatty acid binding protein found in adipocytes. Related to obesity and gestational diabetes.
	fbn2	fbn2	component of connective tissue microfibrils and may be involved in elastic fiber assembly. Related to systemic lupus erythematosus.
	grem1	grem1	BMP antagonist regulating organogenesis, body patterning, and tissue differentiation, especially related to bones.
igf1	igf1	igf1	mediating growth and development
		igj	IgA binding activity and protein homodimerization activity
		kiaa0101	chromatin binding activity involved in centrosome cycle
		kif18b	microtubule depolymerization; mitotic cell cycle; and regulation of cell division
		kif23	cross-bridge antiparallel microtubules and drive microtubule movement
		kif2c	promoting mitotic chromosome segregation.
		lhfpl2	
	lrrc15	lrrc15	collagen binding activity; fibronectin binding activity; and laminin binding activity. Related to cancer metastasis. Data deficiency.
		mex3a	RNA binding activity
		MME	common acute lymphocytic leukemia antigen
		mnd1	DNA recombination and meiotic cell cycle
		neil3	base excision repair
	OLFML2A	***OLFML2A***	extracellular matrix organization. Expressed in cornea, lens, and retina.
	p4ha3	p4ha3	component of prolyl 4-hydroxylase, a key enzyme in collagen synthesis. Related to cancer metastasis. Data deficiency.
		pbk	activation of lymphoid cells and support testicular functions
Pla2g2a	Pla2g2a	Pla2g2a	hydrolysis of the sn-2 fatty acid acyl ester bond of phosphoglycerides, releasing free fatty acids and lysophospholipids
		rrm2	formation of deoxyribonucleotides from ribonucleotides
		Sh3bp5	guanyl-nucleotide exchange factor activity and protein kinase inhibitor activity
		siglec1	positive regulation of T cell apoptotic process and positive regulation of extrinsic apoptotic signaling pathway
		ska1	chromosome segregation; mitotic cell cycle; and regulation of microtubule polymerization or depolymerization
		slc27a6	uptake of long-chain fatty acids
		Sod3	EC SOD
		TMEM100	BMP signaling pathway.
Tnc	Tnc	Tnc	extracellular matrix protein
		top2a	controls and alters the topologic states of DNA during transcription
		tpx2	protein kinase activity; microtubule cytoskeleton organization; and negative regulation of microtubule depolymerization
		ttk	cell proliferation, critical mitotic checkpoint protein
		Tubb5	structural constituent of cytoskeleton, involved in regulation of synapse organization.
		UCHL1	hydrolyzes a peptide bond at the C-terminal glycine of ubiquitin. This gene is specifically expressed in the neurons and in cells of the diffuse neuroendocrine system.
		VEPH1	negative regulation of transforming growth factor beta receptor signaling pathway
